# Three-Dimensional Volumetric Analysis of Frontal Ethmoidal Cells and Evaluation of Influential Factors: A Helical Computed Tomography Study

**DOI:** 10.3390/tomography8060233

**Published:** 2022-11-24

**Authors:** Ana Lúcia Franco Ricardo, Celso Massahiro Ogawa, João Pedro Perez Gomes, Catharina Simioni De Rosa, Sérgio Lúcio Pereira de Castro Lopes, Paulo Henrique Braz-Silva, Kaan Orhan, Andre Luiz Ferreira Costa

**Affiliations:** 1 Postgraduate Program in Dentistry, Dentomaxillofacial Radiology and Imaging Laboratory, Department of Dentistry, Cruzeiro do Sul University (UNICSUL), São Paulo 01506-000, Brazil; 2 Department of Stomatology, School of Dentistry, University of São Paulo (USP), São Paulo 05508-000, Brazil; 3 Department of Diagnosis and Surgery, São José dos Campos School of Dentistry, São Paulo State University (UNESP), São José dos Campos 12245-000, Brazil; 4 Department of Dentomaxillofacial Radiology, Faculty of Dentistry, Ankara University, 06560 Ankara, Turkey

**Keywords:** computed tomography scans, image processing, segmentation, sexual dimorphism, sinus anatomy, three-dimensional reconstruction

## Abstract

In the present study, we aimed to assess frontal ethmoidal cells by using segmentation 3D software to establish a possible correlation between volume variation and both gender and age, as well as a relationship with Keros classification. Helical computed tomography images were obtained from 71 patients for analysis, in which the agger nasi cell, supra agger cell, supra agger frontal cell, supra bulla frontal cell, supraorbital ethmoidal cell, supra bulla cell, and frontal septal cell were identified and segmented to obtain their volume. Significant differences in volume were found for age and gender regarding agger nasi cells (*p*-value = 0.017), supra agger cells (*p*-value < 0.001), and frontal septal cells (*p*-value = 0.049). In the frontal septal cells, an increase of one year in age reduced the volume by 0.309, on average. According to Keros classification, the mean volumes were 10.07 and 25.64, respectively, for types II and III, both being greater than that of type I. Extracting volumetric data by using segmentation software in agger nasi cells, supra agger cells, and frontal septal cells may be useful for obtaining additional information related to age, in addition to possibly contributing to elucidating the anatomical variations in the region and an identification forensic tool.

## 1. Introduction

Knowledge of the anatomy of frontal ethmoidal cells is essential for planning surgical procedures and all sorts of medical treatments in the region. The nasal cavity is defined as a roughly cylindrical, midline airway passage extending anteriorly from the nasal to the choana more posteriorly. The ethmoid sinuses are formed from air cells within the ethmoid bone located between the eyes and the nose [1,2].

Therefore, knowing the anatomical relations of the sinuses with the surrounding structures will help improve the understanding of the paranasal sinus anatomy, which contributes significantly to facilitating medical procedures [3]. In addition, these data may be useful in forensic analysis when a volume difference between men and women is found [4].

In summary, the so-called frontal recess is a varying development space of anterior pneumatized cells [5]. The anatomical knowledge of these structures is of fundamental importance because approaching the frontal recess is considered one of the most difficult procedures in nasosinusal surgery [5,6]. This difficulty is related to several factors, including its dimensions, unfavorable surgical angles, and significant anatomical variability [6]. The frontal recess is limited medially by the middle turbinate, laterally by the lamina papyracea, anteriorly by the frontal apophysis of the upper jaw, and posteriorly by the ethmoidal bulla. This space can be pneumatized by several accessory anterior frontal ethmoidal cells [7,8].

**Figure 1:**
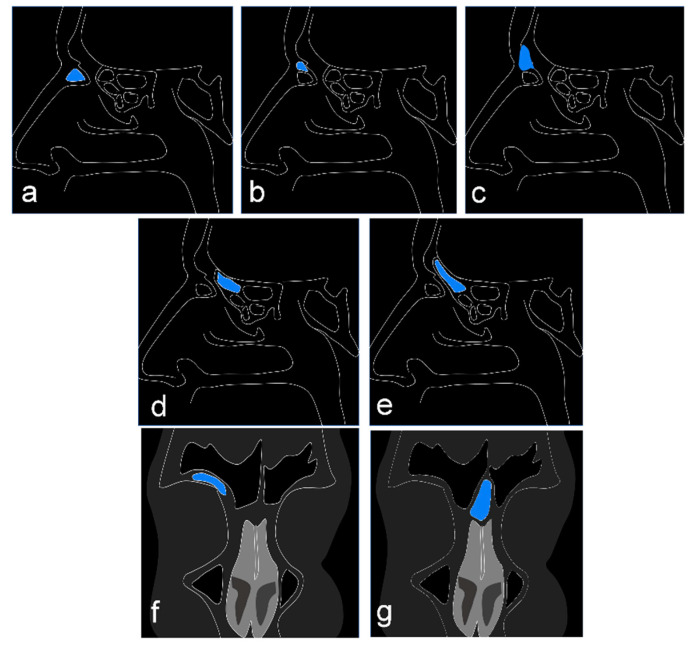
Diagram showing the cell types studied according to the International Frontal Sinus Anatomy Classification. Sagittal CT section: (**a**) cell agger nasi; (**b**) supra agger nasi cell; (**c**) frontal supra agger cell; (**d**) supra bulla cell; (**e**) frontal supra bulla cells. Coronal CT section: (**f**) supraorbital ethmoid cell; (**g**) frontal septal cell.

The frontal ethmoidal cells are categorized according to the International Frontal Sinus Anatomy Classification as follows: agger nasi cell, supra agger cell, supra agger frontal cell, supra bulla frontal cell, supraorbital ethmoidal cell, supra bulla cell, and frontal septal cell [9]. In Figure 1, a diagram shows computed tomography (CT) sections according to the anatomical classification.

The volume assessment of certain structures, such as infra-orbital ethmoidal cells (also known as Haller cells), was already proven to be useful for understanding the anatomical variants of paranasal sinuses [10].

The depth of the olfactory fossa is determined by the height of the lateral lamella of the cribriform plate. According to the Keros classification, the height of the olfactory fossa can be of type I (less than 3 mm), type II (4–7 mm), or type III (8–16 mm) [11,12]. Additionally, a variable part of the lateral wall of the olfactory fossa will be uncovered during dissection of the frontal ethmoidal area [11].

Since the advent of functional nasosinusal endoscopic surgery and computed tomography (CT), the anatomy of the paranasal region has been more closely assessed. In the nasal cavity and paranasal sinuses, there is a multitude of anatomical variants that can interfere with the sinus drainage pathway, leading to infectious processes [11,12]. Some of these anatomical changes are common and can be seen in the majority of the population [12,13]. The failure to identify these anatomical variants has been linked to a higher rate of complications for those patients to undergo functional endoscopic sinus and skull base surgery [14,15]. Understanding the reasons influencing the sizes of these structures is essential in endoscopic sinus surgery [16].

Therefore, the objective of this study was to assess the frontal ethmoidal cells to establish a possible correlation between volume variation and both gender and age as well as to evaluate the relationship between the Keros classification and the frontal ethmoidal cells.

## 2. Materials and Methods

### 2.1. Subjects

The present retrospective study was submitted to and approved by the Research Ethics Committee of the University of São Paulo (USP) according to protocol number 52143121.0.0000.0075. All procedures performed in the study are in accordance with the human research ethical standards set by institutional and/or national research committees and with the 1964 Helsinki Declaration, including later amendments or comparable ethical standards.

The study was carried out by analyzing helical computed tomography images taken between January 2019 and March 2021 and obtained from the database of the Dentomaxillofacial Radiology Department of the School of Dentistry of São José dos Campos, São Paulo State University (UNESP). The CT images were acquired with a 4-channel multi-detector CT scanner (Alexion, Toshiba/Canon, Ohtawara, Japan) without the use of intravenous contrast and along the axial plane, with the patient in the supine position and their head fixed in a neutral position. The imaging protocol was the following: contiguous 1-mm thick slices at 1-mm intervals; operation at 100 kV, 100 mA, 1 s/rotation, matrix of 512 × 512 pixels, a gap of 0.8 mm, voxel size of 0.37 mm × 0.37 mm, and field of view (FOV) of 180 mm × 180 mm; scanning from the nasal process of the maxilla to the apex of the frontal sinus, parallel to the hard palate.

A total of 500 scans were retrieved from the database, all in Digital Imaging and Communications in Medicine (DICOM) format, and two dentomaxillofacial radiologists with 15 years of experience in evaluating CT scans assessed them in consensus.

The CT scans were evaluated based on the following inclusion criteria: male and female patients older than 18 years old and presenting paranasal sinuses completely visible on the CT scan. The exclusion criteria were patients with a history of previous surgery or trauma in the region of the paranasal sinuses, the presence of artifacts (acquisition or patient-related) in the paranasal sinus region, and pathological processes in the paranasal sinuses.

After applying the inclusion and exclusion criteria, the final sample consisted of 71 patients.

### 2.2. Identification and Data Extraction

The DICOM images were analyzed using InVesalius version 3.1 software (http://www.cti.gov.br/invesalius/) (accessed on 10 October 2021) through the manual segmentation of the anatomical structures.

A third dentomaxillofacial radiologist, with extensive knowledge of the tomographic anatomy of the paranasal sinuses, identified and segmented the following structures: agger nasi cell, supra agger cell, supra agger frontal cell, supra bulla frontal cell, supraorbital ethmoidal cell, supra bulla cell, and frontal septal cell. The cells were identified on the right and left sides separately.

**Figure 2:**
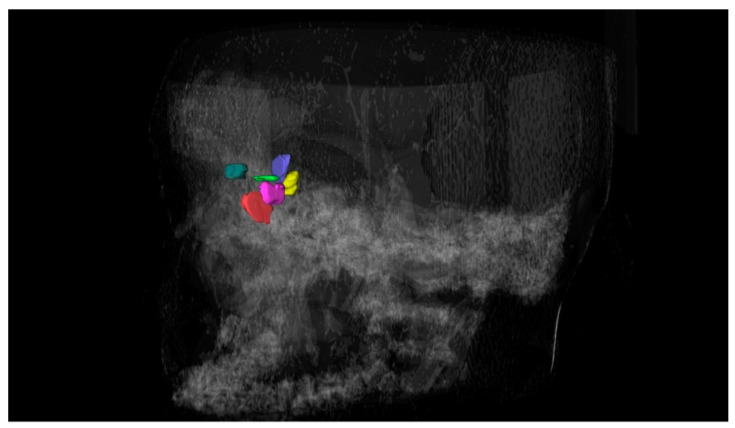
Three-dimensional reconstruction of the frontal ethmoidal cells.

The examiner manually traced the anatomical boundary of the cells on the sagittal, coronal, and axial slices carefully, until the cells were fully segmented. After the segmentation process, a 3D surface representing the topography of each cell was generated (Figure 2) and its volume was automatically calculated by the software based on the limits of the segmented area in the axial, sagittal, and coronal slices. The segmentation process was performed based on previous studies [17], and the volumes were measured in cubic millimeters.

Fifteen days after the evaluations, the intra-rater accuracy was assessed by re-segmenting and re-processing a quarter of the images.

### 2.3. Critical Data Analysis

Exploratory data analysis was performed through summary measures (percentage, mean, standard deviation, median, minimum, and maximum) and chart construction. The intraclass correlation coefficient (ICC) was used to evaluate the repeatability of the volume measurement, whereas Pearson’s correlation coefficient was used to assess the correlation between age and volume. The cell volumes were evaluated on both sides (i.e., right and left) to consider the correlation between the measurements in the same patient. Generalized estimating equation (GEE) models were used to assess the influence of independent variables on the volume, meaning that the *p*-values found were adjusted for correlation between the measurements in the same subject. All statistical analyses were performed at a significance level of 5%.

## 3. Results

A total of 71 patients of both genders (50.1% male) with ages between 18 and 79 years participated in the study. The mean age was 39.6 years old.

The ICC showed an almost perfect intra-rater agreement for the measurements (0.999).

### 3.1. Agger Nasi Cell

A weak negative correlation was observed between the age and volume of agger nasi cells on the right (ρ = −0.171) and left (ρ = −0.212) sides.

**Figure 3:**
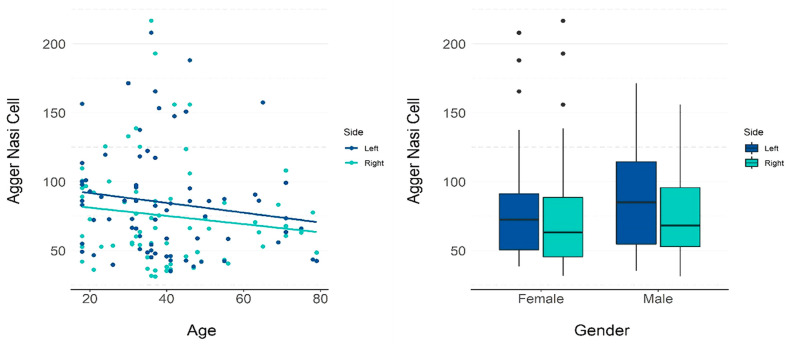
GEE model and scatter plots showing the influence of age and gender on the volume of the agger nasi cell.

Table 1 presents the descriptive measurements of agger nasi cells in terms of volume by gender, and Table 2 and Figure 3 present the *p*-values for the influence of each variable on the volume. Through the regression coefficient, it is noted that an increase of one year in age reduced the volume of the agger nasi cell by 0.327 mm^3^ on average.

### 3.2. Supra Agger Cell

A weak negative correlation was observed between the age and volume of the supra agger cell on the right (ρ = −0.174) and left (ρ = −0.358) sides.

**Figure 4:**
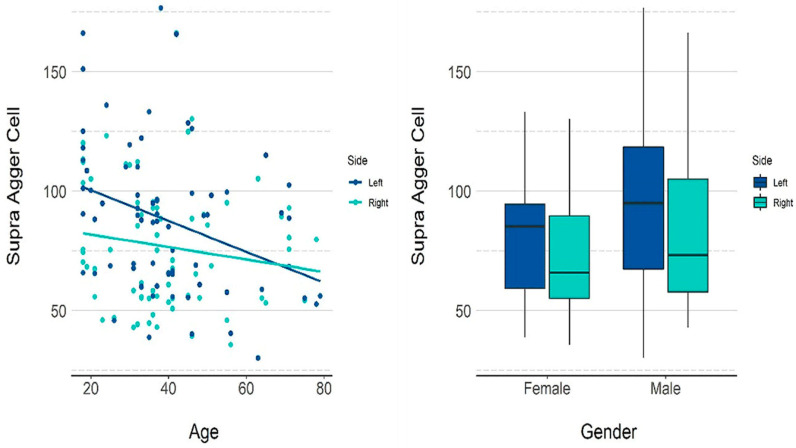
GEE model and scatter plots showing the influence of age and gender on the volume of the supra agger cell.

Table 3 presents the descriptive measurements of the supra agger cell in terms of volume (mm^3^) by gender, and Table 4 and Figure 4 present the *p*-values for the influence of each variable on the volume. Both age and gender had a significant influence on the calculated volume. An increase of one year in age reduced the volume by 0.454 mm^3^ on average. The mean volume in male patients was 14.52 mm^3^ higher than in females.

### 3.3. Frontal Septal Cell

A weak negative correlation was observed between the age and volume of the frontal septal cell on the right (ρ = −0.188) and left (ρ = −0.244) sides.

**Figure 5:**
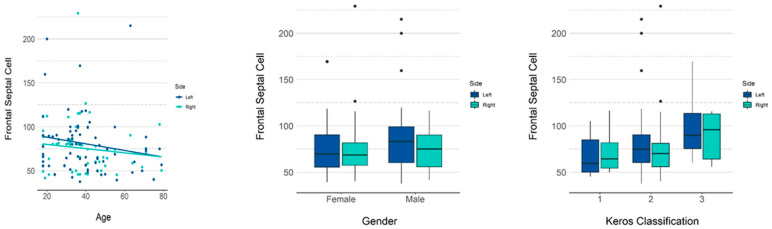
GEE model and scatter plots showing the influence of age and gender and Keros classification on the volume of the frontal septal cell.

Table 5 presents the descriptive measurements of the frontal septal cell in terms of volume (mm^3^) by gender, and Table 6 and Figure 5 present the *p*-values for the influence of each variable on the volume. Among the variables studied, age and Keros classification had a significant influence on volume. An increase of one year in age reduced the volume by 0.309 on average. According to Keros classification, the mean volumes were 10.07 and 25.64, respectively, for types II and III, both being greater than type I.

## 4. Discussion

The anatomy of the paranasal sinuses is one of the most variable in the human body. A systematic review recently highlighted that the most common anatomical variation related to nasal septum deviation includes the agger nasi cell [18]. In the present study, a slightly negative correlation was observed between the age of the patients in our sample and the volume of agger nasi cells on both sides, including the supra agger cell and frontal septal cell. This information implies that as the age increases, the volumes of these three cells slightly decrease.

It is necessary to interpret these data with caution by considering the great anatomical variations of this region, the possible presence of pneumatization, and the low sample size. However, in confirming these results, the volumes of these three cells and their correlation with gender and age can contribute to elaborating treatment plans and, eventually, performing forensic analysis.

Previous studies [19,20] have performed linear measurements of the height and length of agger nasi cells and compared them between males and females as well as between age groups, but no statistically significant relationships have been found between them. The fact that our study was based on a volumetric study may establish a new methodology with a better foundation for new studies using different samples, as the volume values of the supra agger nasi cells showed negative correlations with the ages of the individuals.

For instance, the volume measurements showed that specific regions are larger in male individuals compared to females [21]. Other studies have been performed to estimate the individual’s age based on the pulp chamber volume of the molar tooth [22].

Additionally, a volumetric analysis using a precise segmentation process can eventually contribute to determining anatomical variations, thus facilitating the planning of surgical procedures in this region.

Altıntaş et al. [23], in studying the relationship between the volume of agger nasi cells and the anteroposterior thickness of the frontal recess, concluded that there was no such relationship. However, they emphasized that it is essential to consider the volume of these cells for performing anatomical studies on the drainage complex of frontal sinuses. In our study, we did not take into account other extramural cells other than supra agger nasi cells and frontal supra agger cells. In this way, their results could have been different from those cited, as the presence of these structures would tend to narrow the dimension of the frontal recess. This observation emphasizes the importance of our methodology and results.

In another region of the skull, the olfactory fossa, a statistically significant difference was found in the mean depth between males and females, but not between the left and right sides. The Keros classification type 1 has been found in less than 20% of the cases, whereas type 2 in more than 74%, and type 3 in less than 8%. The authors highlighted that the Keros classification type 3 was more observed on the right side than on the left side, as well as more in men than in women. Asymmetry in the olfactory fossa depth between both sides was seen in 75% of patients [24].

The use of the Keros classification for the study of the olfactory fossa has been cited in the literature, either as an epidemiological measure of its prevalence [24,25,26,27] or with the objective of trying to establish relationships between this classification and anatomical structures and variations [28,29]. In our study, no relationships were identified between the Keros classification and the presence of intra or extramural ethmoid cells, but as mentioned above, type II was the most prevalent, followed by types I and III, which is in agreement with the literature.

This study is important because understanding the possible relationships between pneumatization and the volumes of these cells can help clarify the etiology of sinusitis-related problems. Considering that the cell volume can influence pneumatization, the knowledge of it and its correlations can add important information to the elaboration of treatment plans. It has been described that the morphology of frontal cells is closely related to pathologies involving the frontal recess [30], and pneumatization is surgically important because vascular and nerve structures could be exposed or even compromised if this pneumatization is not considered in the process.

Authors have emphasized that, specifically regarding the olfactory fossa, a preoperative assessment of its depth is extremely important to reduce iatrogenic complications [24]. It has been mentioned that the surgeon’s understanding of the anatomy of the ethmoid roof and its possible variations is of utmost importance for overcoming possible complications during endoscopic sinus surgery [12].

The same reasoning applies to the anatomical variations of the frontal recess. Moreover, the possible correlation between anatomical variations of the sinus region and pathologies of paranasal sinuses has been described in recent studies. However, authors have also highlighted that due to contradictory results in the literature, further research is necessary to elucidate the effects of anatomical variants of the sinus region [14].

In the present study, we aimed to establish a correlation between the volume of the frontal ethmoid sinus and both the age and gender of the subjects. Among all the assessed cells, three have shown statistical significance related to age and gender, namely the agger nasi cell, the supra agger cell, and the frontal septal cell.

It was observed that the volume of the frontal septal cell was greater in cases of Keros classification types II and III, meaning that surgical management of the ethmoidal region could eventually affect it, as there is a relationship between the pneumatization of this cell and the surgical vulnerability of the ethmoidal artery.

However, the present study has some limitations to be considered. The sample size was small, and further studies with an increased number of patients should be carried out. A second limitation is that a single examiner performed the segmentation of the cell images, which might potentially introduce bias.

## 5. Conclusions

Three-dimensional volume segmentation extracted from frontal ethmoidal cells can be useful as an identification forensic tool.

## Figures and Tables

**Table 1:** Position and dispersion measurements of agger nasi cells by gender.

Variable	Side		Mean	Standard Deviation	Minimum	Median	Maximum
Gender	Left	Female	80.9	40.9	38.4	72.4	208.0
		Male	88.5	38.7	35.0	85.0	171.0
	Right	Female	76.5	43.8	31.6	63.1	217.0
		Male	74.0	31.5	31.0	68.1	156.0

**Table 2:** Estimations of the regression coefficients and *p*-values of the GEE model to assess the influence of age and gender on the volume of agger nasi cell.

Variable	Coefficient	*p*-Value
Age	−0.327	0.017
Gender male	2.57	0.69

**Table 3:** Position and dispersion measurements of supra agger cell by gender.

Variable	Side		Mean	Standard Deviation	Minimum	Median	Maximum
Gender	Left	Female	78.2	24.5	38.7	85.1	133.0
		Male	97.0	34.7	30.1	94.9	177.0
	Right	Female	71.5	23.3	35.6	65.8	130.0
		Male	81.7	29.2	42.9	73.3	166.0

Volume (mm^3^).

**Table 4:** Estimations of the regression coefficients and *p*-values of the GEE model to assess the influence of age and gender on the volume of supra agger cell.

Variable	Coefficient	*p*-Value
Age	−0.454	<0.001
Gender Male	14.52	0.002

**Table 5:** Position and dispersion measurements of the frontal septal cell by gender.

Variable	Side		Mean	Standard Deviation	Minimum	Median	Maximum
Gender	Left	Female	74.6	26.3	39.4	69.6	169.0
		Male	86.8	39.0	37.5	83.0	215.0
	Right	Female	75.4	33.2	40.4	68.6	229.0
		Male	75.1	23.5	41.7	75.0	116.0

Volume (mm^3^).

**Table 6:** Estimation of the regression coefficient and *p*-value of the GEE model to assess the influence of age and gender and Keros classification on the volume of the frontal septal cell.

Variable	Coefficient	*p*-Value
Age	−0.309	0.049
Gender Male	5.93	0.250
Keros Classification type I	10.07	0.043
Keros Classification type III	25.64	0.002

## Data Availability

The datasets generated and/or analyzed during the current study are available from the corresponding author upon reasonable request.

## References

[1] Seth N., Kumar J., Garg A., Singh I., Meher R. (2020). Computed tomographic analysis of the prevalence of International Frontal Sinus Anatomy Classification cells and their association with frontal sinusitis. J. Laryngol. Otol..

[2] Choby G., Thamboo A., Won T.B., Kim J., Shih L.C., Hwang P.H. (2018). Computed tomography analysis of frontal cell prevalence according to the International Frontal Sinus Anatomy Classification. Int. Forum. Allergy Rhinol..

[3] Cappello Z.J., Minutello K., Dublin A.B. (2022). Anatomy, Head and Neck, Nose Paranasal Sinuses.

[4] M S., Bagewadi A., Lagali-Jirge V., S L.K., Panwar A., Keluskar V. (2022). Reliability of gender determination from paranasal sinuses and its application in forensic identification-a systematic review and meta-analysis. Forensic. Sci. Med. Pathol..

[5] Park S.S., Yoon B.N., Cho K.S., Roh H.J. (2010). Pneumatization Pattern of the Frontal Recess: Relationship of the Anterior-to-Posterior Length of Frontal Isthmus and/or Frontal Recess with the Volume of Agger Nasi Cell. Clin. Exp. Otorhinolaryngol..

[6] Dassi C.S., Demarco F.R., Mangussi-Gomes J., Weber R., Balsalobre L., Stamm A.C. (2020). The Frontal Sinus and Frontal Recess: Anatomical, Radiological and Surgical Concepts. Int. Arch. Otorhinolaryngol..

[7] Makihara S., Kariya S., Okano M., Naito T., Uraguchi K., Matsumoto J., Noda Y., Nishizaki K. (2019). The Relationship Between the Width of the Frontal Recess and the Frontal Recess Cells in Japanese Patients. Clin. Med. Insights Ear. Nose Throat..

[8] Stammberger H.R., Kennedy D.W., Anatomic Terminology G. (1995). Paranasal sinuses:anatomic terminology and nomenclature. Ann Otol. Rhinol. Laryngol. Suppl..

[9] Wormald P.J., Hoseman W., Callejas C., Weber R.K., Kennedy D.W., Citardi M.J., Senior B.A., Smith T.L., Hwang P.H., Orlandi R.R. (2016). The International Frontal Sinus Anatomy Classification (IFAC) and Classification of the Extent of Endoscopic Frontal Sinus Surgery (EFSS). Int. Forum. Allergy Rhinol..

[10] Friedrich R.E., Fraederich M., Schoen G. (2017). Frequency and volumetry of infraorbital ethmoid cells (Haller cells) on cone-beam computed tomograms (CBCT) of the mid-face. GMS Interdiscip. Plast Reconstr. Surg. DGPW.

[11] Souza S.A., Souza M.M.A.D., Idagawa M., Wolosker Â.M.B., Ajzen S.A. (2008). Computed tomography assessment of the ethmoid roof: A relevant region at risk in endoscopic sinus surgery. Radiol. Bras..

[12] V A.M., Santosh B. (2017). A Study of Clinical Significance of the Depth of Olfactory Fossa in Patients Undergoing Endoscopic Sinus Surgery. Indian J. Otolaryngol. Head Neck Surg..

[13] de Carvalho A.B.G., Ferreira Costa A.L., Fuziy A., de Assis A.C.S., Castro Veloso J.R., Coutinho Manhaes L.R.J., Santamaria M.P., de Castro Lopes S.L.P. (2018). Investigation on the relationship of dimensions of the maxillary sinus drainage system with the presence of sinusopathies: A cone beam computed tomography study. Arch. Oral Biol..

[14] Papadopoulou A.M., Bakogiannis N., Skrapari I., Bakoyiannis C. (2022). Anatomical Variations of the Sinonasal Area and Their Clinical Impact on Sinus Pathology: A Systematic Review. Int. Arch. Otorhinolaryngol..

[15] Fadda G.L., Rosso S., Aversa S., Petrelli A., Ondolo C., Succo G. (2012). Multiparametric statistical correlations between paranasal sinus anatomic variations and chronic rhinosinusitis. Acta Otorhinolaryngol. Ital..

[16] Kaygusuz A., Haksever M., Akduman D., Aslan S., Sayar Z. (2014). Sinonasal anatomical variations: Their relationship with chronic rhinosinusitis and effect on the severity of disease-a computerized tomography assisted anatomical and clinical study. Indian J. Otolaryngol. Head Neck Surg..

[17] Dos Santos W.P., Perez Gomes J.P., Nussi A.D., Joa O.M.A., Botti Rodrigues Dos Santos M.T., Hasseus B., Giglio D., Braz-Silva P.H., Ferreira Costa A.L. (2020). Morphology, Volume, and Density Characteristics of the Parotid Glands before and after Chemoradiation Therapy in Patients with Head and Neck Tumors. Int. J. Dent..

[18] Papadopoulou A.M., Chrysikos D., Samolis A., Tsakotos G., Troupis T. (2021). Anatomical Variations of the Nasal Cavities and Paranasal Sinuses: A Systematic Review. Cureus.

[19] Angelico F.V., Rapoport P.B. (2013). Analysis of the Agger nasi cell and frontal sinus ostium sizes using computed tomography of the paranasal sinuses. Braz. J. Otorhinolaryngol..

[20] Liu Z., Li X., Wang P., Yang G., Li X., Zhao P. (2014). Anatomy and imaging study of a new upper-agger nasi pathway of frontal sinus surgery. Lin Chung Er Bi Yan Hou Tou Jing Wai Ke Za Zhi.

[21] Melke G.S.F., Costa A.L.F., Lopes S., Fuziy A., Ferreira-Santos R.I. (2016). Three-dimensional lateral pterygoid muscle volume: MRI analyses with insertion patterns correlation. Ann Anat..

[22] Ge Z.P., Yang P., Li G., Zhang J.Z., Ma X.C. (2016). Age estimation based on pulp cavity/chamber volume of 13 types of tooth from cone beam computed tomography images. Int. J. Legal. Med..

[23] Altintas A., Celik M., Yegin Y., Canpolat S., Olgun B., Tulin Kayhan F. (2017). Correlation between the extent of pneumatization of Agger agger Nasi nasi cells and the anterior-to-posterior length of the frontal recess: A a computer-assisted anatomical study. Otolaryngol. Pol..

[24] Babu A.C., Nair M., Kuriakose A.M. (2018). Olfactory fossa depth: CT analysis of 1200 patients. Indian J. Radiol. Imaging.

[25] Guven M., Elden H., Yaylaci A., Guven E.M., Kara A., Orha A.T. (2021). Age-dependent differences of the depth of olfactory fossa in children. Braz J. Otorhinolaryngol..

[26] Almushayti Z.A., Almutairi A.N., Almushayti M.A., Alzeadi H.S., Alfadhel E.A., AlSamani A.N. (2022). Evaluation of the Keros Classification of Olfactory Fossa by CT Scan in Qassim Region. Cureus.

[27] Guldner C., Zimmermann A.P., Diogo I., Werner J.A., Teymoortash A. (2012). Age-dependent differences of the anterior skull base. Int. J. Pediatr. Otorhinolaryngol..

[28] Poteet P.S., Cox M.D., Wang R.A., Fitzgerald R.T., Kanaan A. (2017). Analysis of the Relationship between the Location of the Anterior Ethmoid Artery and Keros Classification. Otolaryngol. Head Neck Surg..

[29] Li M., Sharbel D.D., White B., Tadros S.Y., Kountakis S.E. (2019). Reliability of the supraorbital ethmoid cell vs Keros classification in predicting the course of the anterior ethmoid artery. Int. Forum. Allergy Rhinol..

[30] Eweiss A.Z., Khalil H.S. (2013). The prevalence of frontal cells and their relation to frontal sinusitis: A radiological study of the frontal recess area. ISRN Otolaryngol..

